# GSK 650394 Inhibits Osteoclasts Differentiation and Prevents Bone Loss via Promoting the Activities of Antioxidant Enzymes *In Vitro* and *In Vivo*

**DOI:** 10.1155/2022/3458560

**Published:** 2022-09-17

**Authors:** Lin-Yu Jin, Shi-Cheng Huo, Chen Guo, Hai-Ying Liu, Shuai Xu, Xin-Feng Li

**Affiliations:** ^1^Department of Orthopedics, Shanghai Key Laboratory for Prevention and Treatment of Bone and Joint Diseases, Shanghai Institute of Traumatology and Orthopedics, Ruijin Hospital, Shanghai Jiaotong University School of Medicine, Shanghai, China; ^2^Department of Spine Surgery, Peking University people's Hospital, Peking University, Beijing, China; ^3^Department of Orthopedic Surgery, Spine Center, Changzheng Hospital, Navy Medical University, Shanghai 200003, China; ^4^Department of Spine Surgery, Renji Hospital, School of Medicine, Shanghai Jiaotong University, Shanghai, China

## Abstract

Osteoporosis (OP) is one of the most common bone disorders among the elderly, characterized by abnormally elevated bone resorption caused by formation and activation of osteoblast (OC). Excessive reactive oxygen species (ROS) accumulation might contribute to the formation process of OC as an essential role. Although accumulated advanced treatment target on OP have been proposed in recent years, clinical outcomes remain unexcellence attributed to severe side effects. The purpose of present study was to explore the underlying mechanisms of GSK 650394 (GSK) on inhibiting formation and activation of OC and bone resorption in vitro and in vivo. GSK could inhibit receptor activator of nuclear-*κ*B ligand (RANKL-)-mediated Oc formation via suppressing the activation of NF-*κ*B and MAPK signaling pathways, regulating intracellular redox status, and downregulate the expression of nuclear factor of activated T cells c1 (NFATc1). In addition, quantitative RT-PCR results show that GSK could suppress the expression of OC marker gene and antioxidant enzyme genes. Consistent with in vitro cellular results, GSK treatment improved bone density in the mouse with ovariectomized-induced bone loss according to the results of CT parameters, HE staining, and Trap staining. Furthermore, GSK treatment could enhance the capacity of antioxidant enzymes in vivo. In conclusion, this study suggested that GSK could suppress the activation of osteoclasts and therefore maybe a potential therapeutic reagent for osteoclast activation-related osteoporosis.

## 1. Introduction

The dynamic process of regulating bone metabolic homeostasis involves a delicate balance between bone mineral synthesis and resorption, which is mediated by osteoblasts (OB) and osteoclasts (OC) [1]. Abnormal activities of OB and OC could result in development and progression of various bone disorders, such as osteoporosis (OP) [2, 3]. OP is a global skeletal metabolism disorder caused by disruptions in bone mass formation and resorption. The prevalence of osteoporosis is expected to rise exponentially as the global population ages, potentially resulting in an economic and social burden affecting more than 200 million patients [4, 5]. Given that osteoclast plays a key role in bone remodeling, therapeutic methods or drugs, which focus on inhibiting the activity and differentiation of OC, are considered major treatment options for keeping or increasing the bone mass in osteoporosis patients [6, 7].

OC is the only cell type that can promote skeletal bone resorption and origins from hematopoietic cells of the mononuclear macrophage lineage [7]. The differential procedures of OCs are under control by 2-key regulators, macrophage colony-stimulating factor (M-CSF), and RANKL [8, 9]. M-CSF is reponsible for survival and differentiation of OC precursors when by binding with its receptor, c-FMS [10], whereas, RANKL is essential for maturation of OCs and promoting the process of bone resorption [11]. When RANKL binds to its receptor (RANK), TNF receptor-associated factor 6 (TRAF6) is directly recruited, and several intracellular molecular signaling transductions are activated, including the MAPK, NF-B, and PI3K-Akt pathways [12, 13]. The activation of these pathways could directly upregulate the expression of tartrate-resistant acid phosphatase (TRAP), NFATc1, cathepsin K, and c-Fos, which are responsible for initiating the transformation and fusion of OC precursors into mature OCs [14, 15]. Thus, inhibition effects on these pathways may play vital role in treating osteoporosis.

Reactive oxygen species (ROS) and free radicals, caused by RANKL stimulation, are essential for biofunction of OCsy. Antioxidants, such as N-acetylcysteine (NAC), could attenuate the level of ROS and then reduce the number of total OCs [16]. Furthermore, the protective effects of antioxidant molecules, including heme-oxygenase-1 (HO-1), manganese superoxide dismutase (SOD), and various mitochondrial oxidant enzymes, on antioxidative stress have been demonstrated to suppress the osteoclastogenesis via enhancing the cytoprotective enzyme activation [17, 18]. Despite a growing understanding of the downstream targets of ROS, their effects on osteoclastogenesis may be exacerbated by excessive ROS production and redox imbalance through the activation of NF-*κ*B and MAPK signaling pathways. In this regard, regulating oxidative stress may be a promising treatment option for OP [16, 19, 20].

SGK1 (serum- and glucocorticoid-inducible kinase 1) is transcriptionally stimulated by serum and glucocorticoids and belongs to a subfamily of serine/threonine kinases [21]. SGK1 has been reported that it expresses in all mammalian tissues and cells and regulates various cellular behaviors, including proliferation, differentiation, and apoptosis [22–24]. Recently, Zhang et al. [25] showed that SGK1 could promote the breast cancer bone metastasis by upregulating the Orail via NF-*κ*B signaling pathway and was essential for osteoclastogenesis. Consequently, inhibition of SGK1 could be a potential target of osteoporosis. GSK 650394 (GSK) was a highly selected inhibitor of SGK1 according to previous studies [26, 27], and the application of GSK could suppress the activation of osteoclast [25]. However, the underlying antiosteoclastogenesis mechanisms of GSK remain unclear. Here, we examined the effects of GSK on osteoclastogenesis, bone resorption, and osteoclast-related gene expression and investigated underlying mechanism of GSK on ROS and RANKL-mediated signaling pathways in vitro. Furthermore, we found that GSK could prevent bone mass loss and enhanced the activity of antioxidants in animal osteoporosis model (ovariectomized, OVX) in vivo, which indicated potential therapeutic application of GSK in osteoporosis.

## 2. Materials and Methods

### 2.1. Reagents and Materials

GSK was acquired from MedChemExpress (MCE, USA) and 10 mM of it was then dissolved in DMSO. We obtained M-CSF and RANKL (both mouse recombinant proteins) from Bio-Techne (USA). Gibco provided the fetal bovine serum (FBS) and *α*-minimal essential medium (*α*-MEM) (ThermoFisher, USA). We purchased penicillin and streptomycin from Fushen Biotech (Shanghai, China). We bought the trap stain kit from Sigma-Aldrich (USA). Fushen Biotech provided phalloidin that had been FITC-labeled (Shanghai, China). Invitrogen provided a LIVE/DEAD (viability/cytotoxicity) kit for mammalian cells (Carlsbad, CA, USA). All PCR-related equipment, including the RNA extraction kit, was bought from Takara (Japan). Primary and secondary antibodies were purchased from Cell Signaling Technology (MAPK family antibody sampler kit: #9926; phospho-MAPK family antibody sampler kit: #9910; NF-*κ*B pathway antibody sampler kit: #9936, USA) for the following targets: p-ERK, p-JNK, p-p38, p38, p-Ikk, p65, IkB, NFATc1, and GAPDH. Proteintech was where the other principal antibodies, such as HO-1, CAT, and GSR, were bought (Wuhan, China). The test kits for the antioxidant enzyme activity (T-AOC, SOD, CAT, and GSSG/GSH) were purchased from Beyotime (Shanghai, China). Shanghai Fushen Biotech Co., Ltd. provided H&E staining kit (Shanghai, China). We bought PMSF and RIPA lysis buffer from Beyotime (Shanghai, China). Additionally, a kit for measuring reactive oxygen species was acquired from Beyotime, and the cell lines MC3T3-E1 and HEPG2 were purchased from the Chinese Academy of Science cell bank (Shanghai, China).

### 2.2. Mouse Bone Marrow Macrophages (BMMs) Culture and Osteoclast Differentiation

Mouse bone marrow macrophages were extracted from the femur and tibia and cultured in 10% FBS supplemented to ɑ-MEM (containing 1% penicillin/streptomycin and 50 ng/mL M-CSF), as previously described [28]. The anchorage-dependent cells continued to develop in an incubator at 37°C and 5% CO2 until they reached 90% confluence, and the suspension cells were removed. To start the OC differentiation, BMMs were placed into twenty-four-well plate with 1.0 × 10^5^ cells in each well and treated using ɑ-MEM (10% FBS, containing 1% penicillin/streptomycin and 30 ng/mLM-CSF, 50 ng/mL RANKL) with the presence of various doses of GSK (0, 1, 2, and 5 *μ*M). BMMs were cultured for five days, and the culture medium was changed every other day. Furthermore, the BMMs only treated with M-CSF were considered as negative controls. Then, plates were washed using PBS for three times. Paraformaldehyde (4%) was used to fix these cells for 15 mins, and TRAP staining kit was used to label the OCs. After staining, we randomly chose three images of separate view fields to count the TRAP-positive cells (mature OC has more than 3 nuclei) of per fields and analyze the percentage of OC area.

### 2.3. Cell Viability

The primary BMMs, osteoblast cell line (MC3T3-E1), and HEPG2 were used in this section to test the cytotoxic effects of GSK using a CCK-8 kit. In brief, each kind of cell lines were seeded at 1.0 × 10^4^ cells per well in a 96-well plate and cultured in ɑ-MEM (10% FBS, containing 30 ng/mLM-CSF) and different doses of GSK (0.1, 0.2, 0.5, 1, 2, 5, 10, 40, and 80 *μ*M). After twelve hours, twenty-four hours, seventy-two hours, or one hundred and twenty hours, each well was filled with CCK-8 solution (v/v: 1/10), and the plates were then incubated at 37°C for two hours. Finally, using a microplate reader, the OD values were determined at 450 nm (Bio-Tek, USA). Moreover, live/dead staining was used to determine cell viability, which was treated with GSK at 5 *μ*M. Briefly, the cells were treated with GSK for 48 hours and subsequently washed three times with PBS. The LIVE/DEAD staining kit was then added to each plate and incubated for fifteen mins at room temperature. Finally, results were obtained using a fluorescence microscope (FluoCa, BioHD, Shanghai, China).

### 2.4. Immunofluorescence Staining for F-Actin Formation

BMMs were seeded into ninety-six-well plates and incubated with various doses of GSK while being exposed to m-CSF and RANKL for a total of 6 days, and the primary cell concentration was 1 × 10^4^. Additionally, untreated BMMs (M-CSF was the only treatment) were used as a negative control. Then, the treated OCs were fixed using 4% paraformaldehyde for 15 mins. After being washed with PBS for three times, 0.2% Triton X-100 was used to permeabilized for 10 min. PBS was used to wash triple time, and FITC-labeled phalloidin (1 : 200) was used to stain these cells at 37°C for 15 mins in the dark. Finally, DAPI staining kit was used to label the nuclei for 5 mins. After washed with PBS for three times, the plates were visualized with a fluorescence microscope (FluoCa, BioHD, Shanghai, China).

### 2.5. Bone Resorption Pit Assay

Bone resorption test was performed according to our previous study [28]. Briefly, 96-well plates with preloaded bovine bone discs were used to seed BMMs with 1.0 × 10^4^ cells per well. Cells were treated in normal culture medium with M-CSF (30 ng/mL) and RANKL (50 ng/mL) for three days. At the same time, GSK was then administered at different dosages of 1, 2, and 5 M. In addition, untreated BMMs (only M-CSF treated) served as negative controls. After OC formation and removing the adherent cells using ultrasonic cleaner, the bone disc was fixed with 2.5% glutaraldehyde for 1 hour, and then, Hitachi scanning electron microscopy (S-4800, Tokyo, Japan) was used to capture images of pits caused by mature OCs. The areas of resorption pits were measured by image J software.

### 2.6. RNA Isolation and Quantitative RT-PCR

BMMs were treated with full medium combining with M-CSF, RANKL, and serial dilutions of GSK (0, 1, 2, and 5 *μ*M) for 5 days or 1, 3, or 5 days. Trizol (Invitrogen) was used to extract total RNA in a volume of 500 *μ*L, and Prime Script RT kit was used to synthesize the cDNA. The qRT-PCR was performed at least triple times using ABI Prism 7500 (Norwalk, USA). The 2^-*ΔΔ*CT^ method was performed and calculated to analyze results in which data were normalized using the relative expression of GAPDH as control. The primer sequences used in present study for RT-PCR could be seen at Table 1.

### 2.7. Western Blot Analysis

BMMs were placed into six-well plates with 1 × 10^6^ cells per well, co-treated with/without GSK, and then stimulated with RANKL to assess the expression of antioxidant enzymes and signaling proteins impacted by GSK. To extract the total proteins from the plates, we used RIPA lysis buffer, 5 × loading buffer was diluted to 1 × loading buffer with protein lysates. After SDS-PAGE gel separation, lysates were transferred to PVDF membranes (Milipore). Following that, the membranes were blocked using nonfat milk and then incubated with primary antibodies in 2% BSA at 4°C overnight. After that, incubation with the second HRP-antibody was carried out for 2 hours after washing the blocked membranes with TBST for 3 times. Antibody reactivity was detected using Bio-Rad imaging system according to the manufacturer' instructions.

### 2.8. Measurement of ROS Levels in Cells

The ROS levels in cells were investigated using DCF-DA according to manufacturer recommendations of the kit. In brief, BMMs treated with only M-CSF were considered as negative control (-RANKL). Cells treated with M-CSF and RANKL were considered as positive controls (-GSK), whereas BMMs treated with RANKL and M-CSF at the presence of GSK (2, 5 *μ*M) were considered as treatment groups. All groups were incubated in 10 *μ*M DCF-DA for 1 hour at 37°C. DAPI was used to stain nucleus. The fluorescence indicating the ROS level was detected using Leica confocal microscope. The intensity of ROS-positive cells was analyzed in each view using Image J software.

### 2.9. In Vivo Animal Studies for Osteoporosis

Eight-week-old C57BL/6 mice were randomly divided into 4 groups (*n* = 5 for each group): Sham (only injected with saline), vehicle (OVX injected with saline), low-dose GSK (OVX injected with 10 mg/kg GSK), and high-dose GSK (OVX injected with 30 mg/kg GSK injection). Mice were subjected to bilateral OVX or Sham surgery after a one-week adaptation feeding period. In brief, the skin was dissected bluntly until it reached the abdominal cavity after a 1-cm dorsal midline incision. The ovary's protective adipose tissue in the abdominal cavity was seized and removed. Once the ovary was located, the uterine horns and vessels 0.5–1 cm in front of it were tied off. The remainder of the tissue was then reinserted into the abdomen after the ovary and ligated adipose tissue had been removed. On sham-operated mice, the same surgical technique was carried out, with the exception of ovaries ligation and removal. GSK supplementation was based on intraperitoneally administration three times a week and a total for 8 weeks. At the end of eighth week, all mice were sacrificed using over dose pentobarbital to collect the tibial bone for micro-CT, histological analysis, and antioxidant enzyme activity test. Furthermore, the major organs, such as the heart, liver, spleen, lung, and kidney, were preserved for HE staining. All animal experimental procedures were reviewed and approved by the Animal Ethical Committee of Peking University People's Hospital.

### 2.10. Micro-CT Scanning

The micro-CT was used to analyze the microstructure changes in the tibial bone according to previous study [28]. In brief, the fixed tibias were scanned using *μ*CT (Bruker micro-CT; 80Kv, 112 mA, equidistant resolution 20 *μ*m, exposure time 300 ms). The quantitative assessment and parameters, like bone volume/tissue volume (BV/TV), bone mineral density (BMD), trabecular number (Tb.N), trabecular thickness (Tb.Th), trabecular separation (Tb.Sp), connectivity density (Conn.Dn), and structure model index (SMI), were calculated using a scanner software according to the software's instructions.

### 2.11. Histological Analysis

The histological detection was performed according to previous study [28]. All fixed tibias were decalcified and embedded into paraffin, and sections were obtained via a 4 *μ*m microtome. Subsequently, the sections were stained with hematoxylin and eosin (HE) and TRAP assay kit. Finally, the quantitative parameters include the ratio of osteoclast surface to bone surface (Oc.S/BS).

### 2.12. Measurement of Antioxidant Enzymes in Tibia

The fresh tibia tissue was ground at the presence of liquid nitrogen and made into protein homogenate, followed with centrifuging at 3000*g* for 15 min. The antioxidant enzyme activity kits were used to test the activities of total antioxidants (T-AOC), catalase (CAT), superoxide dismutase (SOD), oxidized glutathione (GSSG), and glutathione (GSH). All procedures were performed according to the manufacturer's instructions.

### 2.13. Statistical Analysis

Data are displayed as means ± standard deviation (SD) of three or more independent replicates. Differences between groups were assessed by one-way analysis of variance (ANOVA). *P* values <0.05 (*P* < 0.05) were considered statistically significant.

## 3. Results

### 3.1. The Concentrations of GSK Used in This Research Are Safe for Mammalian Cells (BMMs, HEPG2, and MC3T3-E1)

**Figure 1:**
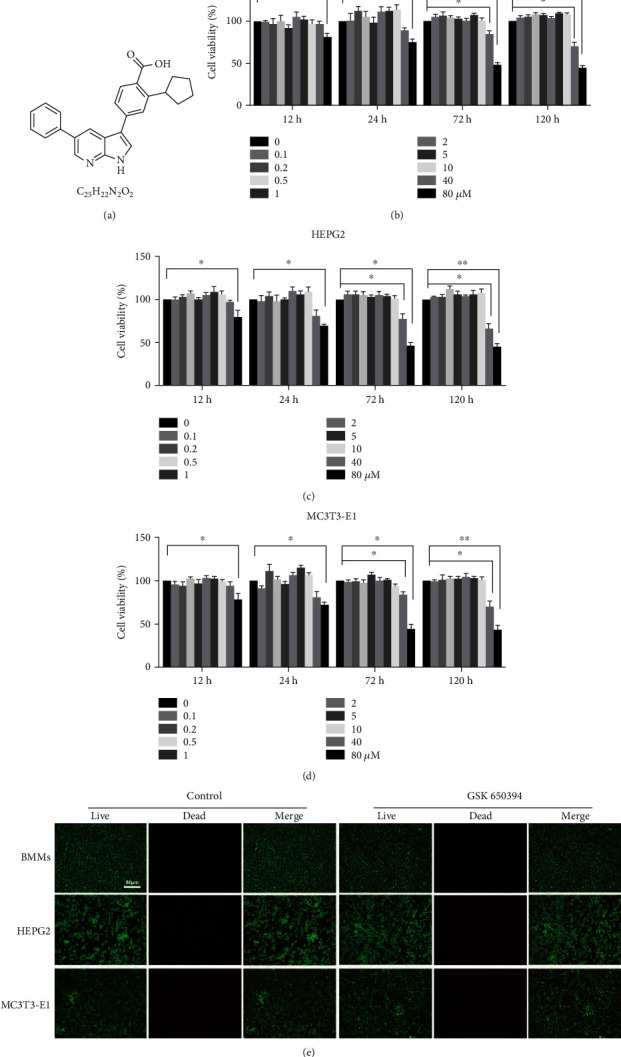
The cytotoxicity of GSK at determined concentrations did not suppress the viability of osteoclast precursor cells. (a) The structure of GSK. (b–d) The viability of BMMs, HEPG2, and MC3T3 E1 treated with different levels of GSK for 12 hours, 24 hours, 48 hours, 72 hours, and 120 hours, as tested by CCK-8 assay. (e) Fluorescence microscopy images of BMMs, HEPG2, and MC3T3-E1 cells stained by LIVE/DEAD Kit after 3 days treatment with GSK. Calcine-AM (green) is used to stain live cells, while ethidium homodimer is used to stain dead cells (red color) scale bar: 50 *μ*m. Data are presented as means ± SD, *n* = 3 (^∗^*P* < 0.05, ^∗∗^*P* < 0.01).

The cytotoxicity of GSK was detected using CCK-8 and LIVE/DEAD staining methods. The viability of cell lines (BMMs, HEPG2, and MC3T3-E1) was cultured in different concentrations of GSK. As shown in Figures 1(b) and 1(c), the range of cytotoxic responses is various for these three cell lines. GSK at a dosage of 5 M did not have any discernible cytotoxic effects on any of the three types of cells, even after treatment extended to 5 days. For BMMs, GSK cytotoxic was obvious at the concentration up to 80 *μ*M at 12 and 24 hours and 40 *μ*M at 72 and 120 hours. As for HEPG2 and MC3T3-E1, there were no obvious differences in cellular viability compared to the control group. Furthermore, LIVE/DEAD staining was used to determine the viability of the three kind mammalian cells treated with GSK at concentration of 5 *μ*M. As shown in Figure 1(e), low level of cell death is determined in the treatment groups and is similar with that in control groups. Taken together, these results demonstrated that the doses of GSK we used *in vitro* were in the safe range for mammalian cells.

### 3.2. GSK Inhibits RANKL-Mediated OCs Formation and OC-Associated Bone Resorption

**Figure 2:**
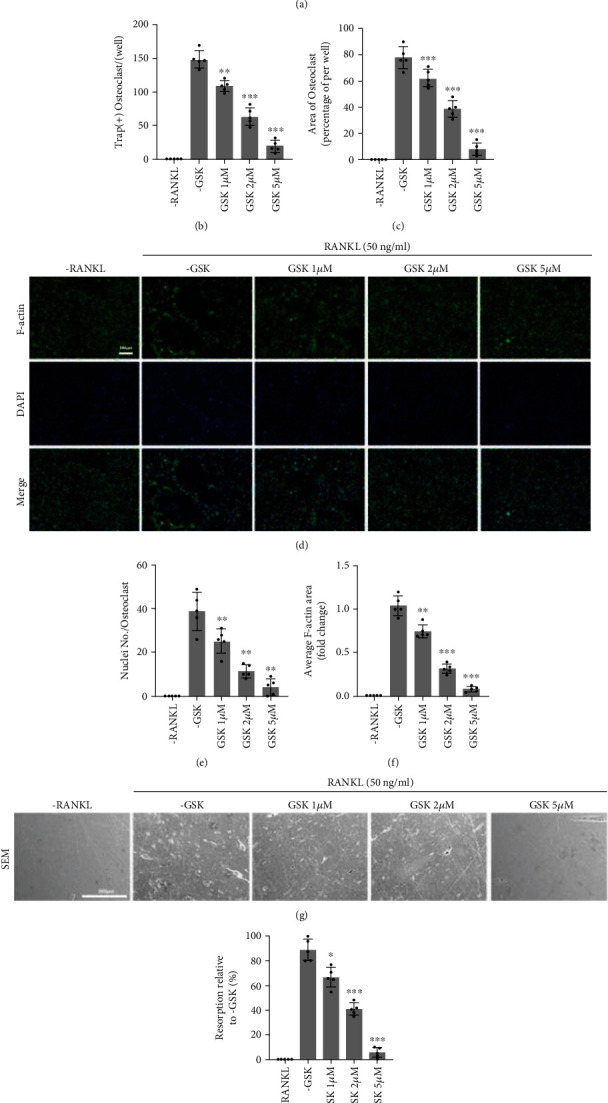
GSK inhibited osteoclast differentiation in vitro in a concentration-dependent manner. BMMs were given m-CSF (50 ng/mL) and RANKL (50 ng/mL) in combination with various GSK concentrations (0, 1, 2, and 5 M). Furthermore, BMMs treated only with m-CSF served as a negative control group. (a) TRAP staining. Scale bar: 200 *μ*m. (b–c) Quantitative on the number of multinuclear cells and area of mature osteoclast. (d) F-actin staining using FITC-phalloidin and cell nuclei staining with DAPI. Scale bar: 200 *μ*m (e–f). (g) Bone disc resorption pits scanned using SEM. Scale bar: 200 *μ*m. (h) Quantitative areas of bone resorption were analyzed using Image J software. Data are presented as means ± SD, *n* = 5 (^∗^*P* < 0.05, ^∗∗^*P* < 0.01, ^∗∗∗^*P* < 0.001).

To analyze whether GSK could inhibit RANKL-mediated OC differentiation, TRAP staining (specific-characteristic of OC) was adopted to study the suppression effects of GSK on RANKL-mediated OC formation at a range of concentrations (1, 2, 5 *μ*M). As shown in Figures 2(a)–2(c), the BMMs differentiation is significantly suppressed by GSK. Furthermore, the ratio of multinuclear mature OCs was reduced in a dose-dependent manner compared to the -GSK group and -RANKL group.

As shown in Figures 2(d)–2(f), FITC-labeled phalloidin is used to mark the mature OCs. The -GSK group exhibited clearly defined rings, and GSK suppressed osteoclastic and actin rings formation. In addition, bone resorption was an important function of mature OCs during the trabecular and cortical mineral remodeling process. Therefore, we use bovine bone discs to determine the resorption capacity of mature OCs. As seen in Figures 2(g) and 2(h), obvious bone resorption pits could be seen in the group treated without GSK. The resorption pits decreased markedly with GSK treatment, especially when it was applied at a concentration of 5 *μ*M. Collectively, these data demonstrated that GSK significantly suppressed the osteoclastogenesis induced by RANKL and inhibited OC-related bone resorption activity in vitro.

### 3.3. GSK Inhibited ROS Levels by Promoting Antioxidant Enzymes Expression *In Vitro*

**Figure 3:**
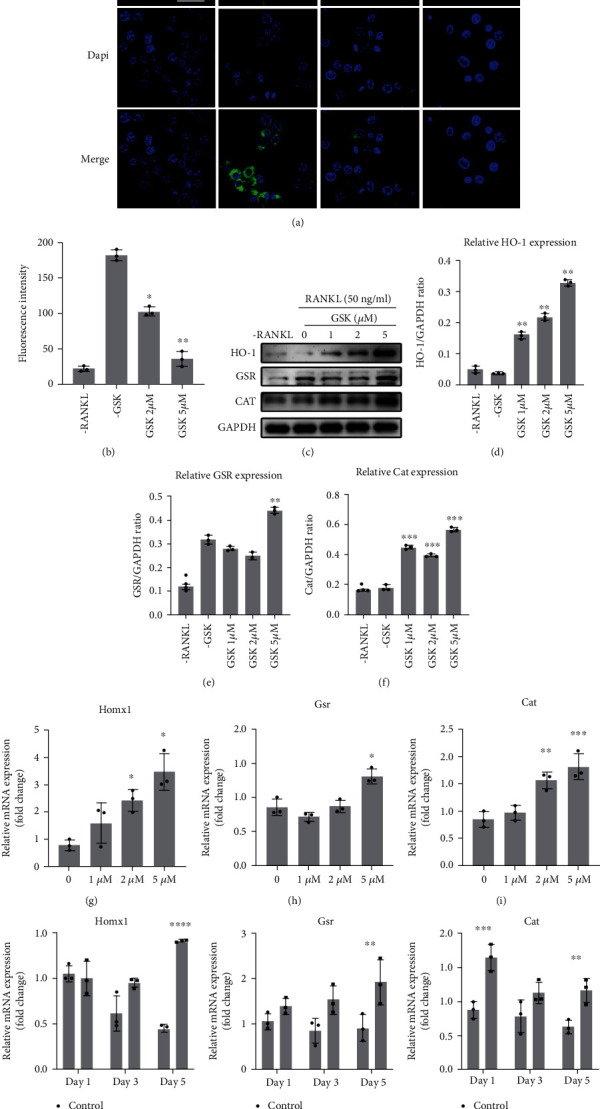
GSK regulates level of reactive oxygen species (ROS) and activities of antioxidant enzymes in vitro. (a) ROS levels in BMMs using DCF-DA. Scale bar: 20 *μ*m. (b) Quantitative analysis of DCF fluorescence intensity. (c) Representative images protein expression of HO-1, GSR, and CAT. (d–f) Quantitative analysis of protein intensity of HO-1, GSR, and CAT relative to *β*-actin. (g–i) Antioxidant enzyme genes of BMMs treated with M-CSF and RANKL with/without different GSK concentrations (0, 1, 2, or 5 *μ*M) for 5 days. (j–l) Antioxidant enzyme genes of BMMs treated with or without 5 *μ*M GSK, for 1, 3, or 5 days, respectively. Data are expressed as the mean ± SD, *n* = 3. ^∗^*P* < 0.05, ^∗∗^*P* < 0.01, ^∗∗∗^*P* < 0.001, ^∗∗∗∗^*P* < 0.0001 versus -GSK group. Abbreviations: CAT (Cat): catalase; GSR (Gsr): glutathione-disulfide reductase; HO-1 (Homx1): heme oxygenase; ROS: reactive oxygen species; BMMs: bone marrow macrophages.

The DCFH-DA fluorescence probe reagent was used to evaluate the ROS level in BMMs treated with RANKL and/or GSK. Intracellular ROS level was the highest in the -GSK-treated group compared to -RANKL group and GSK groups (Figures 3(a) and 3(b)). With the presence of GSK, the dichlorodihydrofluorescein (DCF) intensity was significantly decreased at the concentration of GSK at 5 *μ*M. Furthermore, several antioxidant enzymes were tested as well to determine if GSK could decrease intracellular ROS levels by regulating antioxidant enzymes. As a result of RANKL stimulation, HO-1 and catalase (CAT) expression were partially reduced, but glutathione-disulfide reductase (GSR) expression was significantly increased. Furthermore, GSK could promote the genes expression of these three antioxidant enzymes (Figures 3(g)–3(l)). Taken together, GSK treatment could enhance the expression of these enzymes at the concentration of 5 *μ*M, and our data demonstrated that GSK suppressed RANKL-mediated intracellular ROS levels via promoting antioxidant enzymes expression.

### 3.4. GSK Inhibited OC-Specific Gene Expression

**Figure 4:**
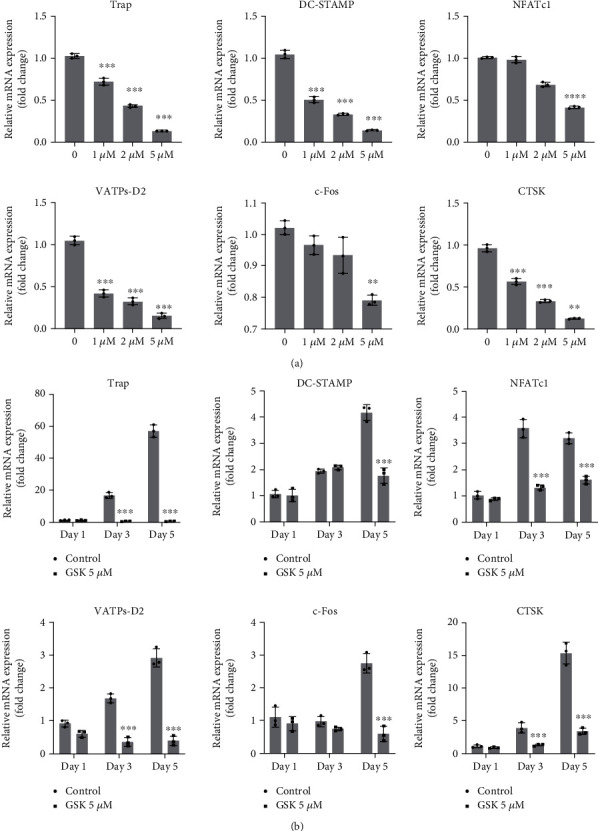
Osteoclastic marker genes could be inhibited by GSK. (a) Osteoclastic marker genes of BMMs treated with M-CSF and RANKL with/without different GSK concentrations (0, 1, 2, or 5 *μ*M) for 5 days. (b) Osteoclastic marker genes of BMMs treated with or without 5 *μ*M GSK, for 1, 3, or 5 days, respectively. Data are expressed as the mean ± SD (*n* = 3), ^∗^*P* < 0.05, ^∗∗^*P* < 0.01, ^∗∗∗^*P* < 0.001 versus -GSK group.

Stimulation of RANKL was known to upregulate several specific osteoclatogenesis-related genes. Hence, we tested several key genes, including CTSK, c-Fos, VATPs-d2, NFATc1, and TRAP via qPCR method. As shown in Figure 4, treatment with GSK obviously suppresses the expression level of osteoclastogenesis-related gene induced by RANKL.

### 3.5. GSK Suppressed NF-*κ*B, MAPK, and NFATc1 Activation

**Figure 5:**
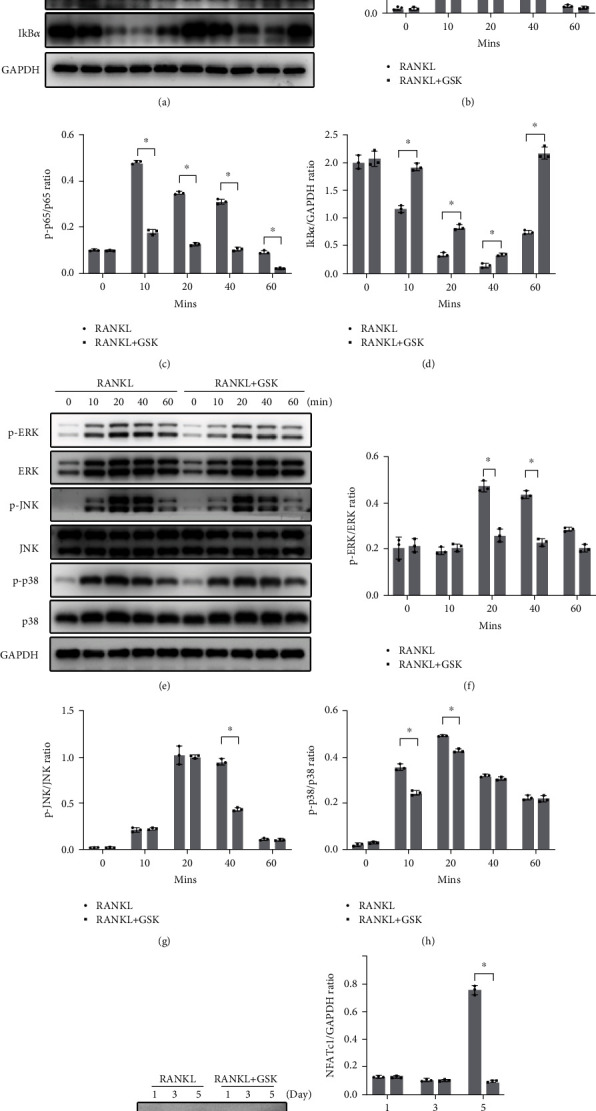
GSK inhibited the activation of NF-*κ*B, MAPK, and NFATc1 signaling pathways during the procedure of osteoclastic differentiation. (a) Average intensity ratio of phosphorated-Ikk*β* to Ikk*β*, phosphorated-p65 to p65, and IkB*α* to GAPDH in BMMs pretreated with/without 5 *μ*M GSK and stimulated by RANKL after 10, 20, 40, and 60 mins. (b–d) Quantitative of protein intensity. (e) Average intensity ratio of phosphorated-ERK to total ERK, phosphorated-JNK to total JNK, and phosphorated-p38 to total p38 in BMMs at the presence of 5 *μ*M GSK or not and stimulated by RANKL after 10, 20, 40, and 60 mins. (f–h) Protein intensity was analyzed using Image J software. (i) Expression level of NFATc1 in BMMs at the presence of 5 *μ*M GSK for 1, 3, or 5 days. (j) Protein intensity for NFATc1 was quantified using Image J software. *n* = 3, ^∗^*P* < 0.01 vs control.

The activation of RANKL-mediated signal transductions is essential for the OC differentiation process, and NF-*κ*B pathway is one of the several cascades activated by RANKL on BMMs precursors during the osteoclastogenesis procedures [7, 12]. Therefore, western blotting was employed to analyze the effects of GSK on the NF-*κ*B signaling pathway activated by RANKL in this study. Here, we found that GSK could attenuate phosphorylation of Ikk*β* and p65 induced by RANKL and furthermore delay degradation of I*κ*B-*α* (Figure 5). The activation phosphorylation of Ikk*β* could lead to the degradation of I*κ*B-*α*. As soon as the degradation happened, the phosphorylation of p65 was activated and then translocated from cytoplasm to the nucleus, where p-p65 could target on several OC-related specific genes and exerted the transcriptional function. Treatment with GSK (5 *μ*M) suppressed the degradation of I*κ*B-*α* and attenuated the phosphorylation of Ikk*β* and p65. The MAPK transduction signal is another important pathway for the OCs differentiation [7, 16]. In line with NF-*κ*B signaling, RANKL-mediated cascades also contained all 3 members of the MAPK signal (ERK, JNK, and p38). The activation of phosphorylation ERK, JNK, and p38 also resulted in the activation of downstream OC-related genes. Here, pretreatment of BMMs with GSK significantly suppressed the phosphorylation of ERK, JNK, and p38. NFATc1 was the master transcriptional activator for OC formation [29]. The activity of NFATc1 not only depends on NF-*κ*B signaling pathway, but also is regulated by MAPK signal transduction. As shown in Figure 5, the expression level of NFATc1 was significantly decreased after treating with GSK at a dose of 5 *μ*M. Collectively, present results showed that GSK could inhibited RANKL-stimulated NF-*κ*B, MAPK, and NFATc1 activation.

### 3.6. GSK Prevented Ovariectomy-Induced (OVX) Bone Loss via Regulating the Redox Balance *In Vivo*

**Figure 6:**
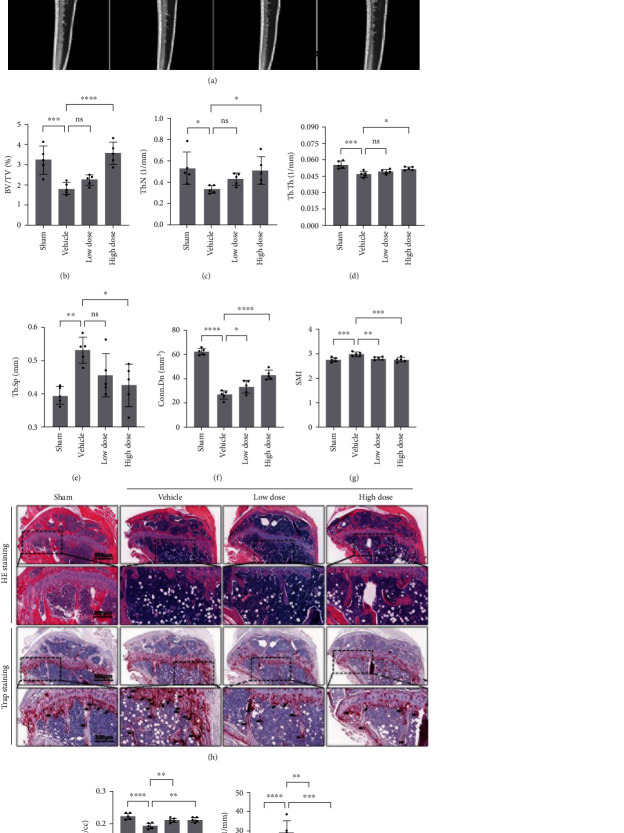
GSK suppresses bone loss of the ovariectomy mice *in vivo*. (a) Reconstruction of tibia using *μ*CT for the sham mice, OVX with saline (vehicle), OVX with 10 mg/kg GSK (low dose), and with 30 mg/kg GSK (high dose) (b–g) Quantitative analysis of bone volume/tissue volume (BV/TV), trabecular number (Tb.N), trabecular thickness (Tb.Th), trabecular separation (Tb.Sp), connectivity density (Conn.Dn), and structure model index (SMI). (h) HE and TRAP staining were used to determine bone architecture and osteoclast activity, respectively (the black arrows represent osteoclasts), scale bar: 500/200 *μ*m. (i–j) The counted number of osteoclasts per sections (N.Oc/BS) and the ratio of osteoclast surface/bone surface (Oc.S/BS). Data are presented as the mean ± SD (*n* = 5), ^∗^*P* < 0.05; ^∗∗^*P* < 0.01; ^∗∗∗^*P* < 0.001; ^∗∗∗∗^*P* < 0.0001.

**Figure 7:**
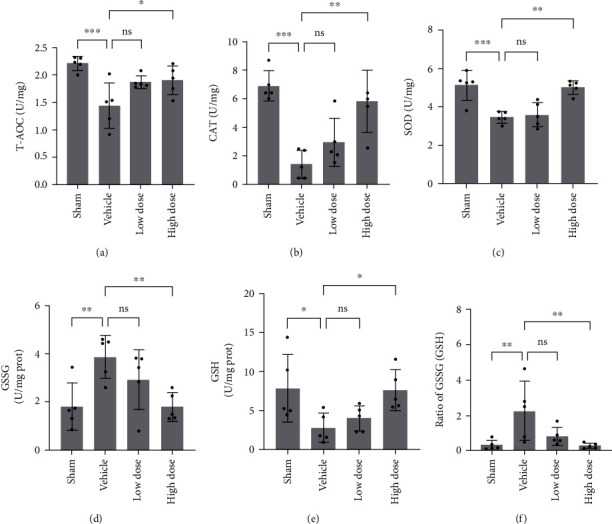
GSK alleviates oxidative stress associated with OVX in vivo. (a) Total antioxidants (T-AOC), (b) catalase (CAT), (c) superoxide dismutase (SOD), (d) oxidized glutathione (GSSG), (e) glutathione (GSH), and (f) ratio of GSSG/GSH, (*n* = 5), ^∗^*P* < 0.05; ^∗∗^*P* < 0.01; ^∗∗∗^*P* < 0.001; ^∗∗∗∗^*P* < 0.0001.

To explore whether GSK possess the therapeutic ability on bone mass remodeling, OVX mice model treated with 10 mg/kg as low dose and 30 mg/kg as high dose was used. After 8-week supplementation of GSK, mouse tibias were collected and analyzed bone mass using micro-CT (Figure 6(a)). As expected, OVX caused trabecular bone loss in OVX mice when compared to the Sham group. The bone density-related parameters (BV/TV, Tb.N, Tb.Th, BMD, and Conn.Dn) were significantly decreased, and the parameters of Tb. Sp and SMI were increased in the OVX group (Figures 6(b)–6(g)), while these trends were dose-dependently reversed by GSK supplementation. Histologic and histomorphometric analyses were conducted to study GSK's protective effects on bone mass loss. In accordance with micro-CT results, GSK prevented bone mass loss when compared to OVX group (Figure 6(h)). The improvements after GSK treatment were due to decreased OC activity around the bone surface and a significant decrease in the total number of TRAP-positive OCs at the tibial bone (Figures 6(i) and 6(j)). The redox balance of bone tissue could be disturbed by the reduction of estradiol level in OVX mice. To identify the effects of GSK on the redox status of tibia, we tested several protein markers of redox enzymes, such as T-AOC, SOD, CAT, GSH, and GSSG. T-AOC, SOD, and CAT capacity were all reduced as a direct result of OVX (Figure 7). Upon treatment with GSK, T-AOC and SOD capacity could be reversed, dose-dependently. Redox balance of tissue was determined by the level of oxidized glutathione (GSSG) and reduced glutathione (GSH). Here, in OVX mice, there was an obvious consumption of GSSG and a significant accumulation of GSH, demonstrating that OVX led to redox unbalance compared to Sham group, while GSK could restore the redox balance (Figure 7). Simultaneously, no visible damage to major organs such as the heart, liver, spleen, lung, or kidney was observed (Figure S1), indicating that GSK exhibited no apparent toxicity in vivo. Based on these findings, GSK could potentially be a potential osteoporosis therapeutic molecule by restoring the redox balance and preventing bone loss.

## 4. Discussion

**Figure 8:**
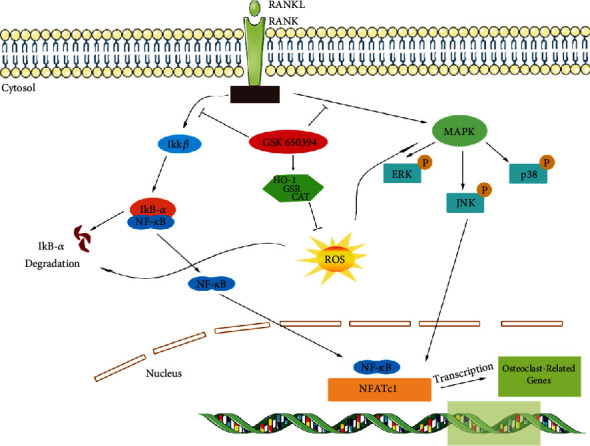
Schematic diagram for the underlying mechanism of GSK in RANKL-induced osteoclastogenesis.

Loss of bone mass, deterioration of bone microstructure, and an increase in bone fragility and fracture are all symptoms of the bone metabolism condition osteoporosis [30]. As the world's aging population keeps growing, osteoporosis-related fractures have emerged as a common cause of illness and mortality [4]. Excessive activation of OCs differentiation is the major contributor for age-related osteoporosis [7]. Until now, several drugs, such as hormone replacement therapy, bisphosphonates, RANKL inhibitors, and teriparatide, have been developed to treat osteoporosis via preventing OC-mediated bone resorption [31–34]. However, side effects of these commercialized drugs also have been reported, such as embolism, breast tenderness, and jaw osteonecrosis [34–37]. Thus, it is important to explore new tolerable and multifunctional therapeutic agents. Here, we showed that the GSK dose used in vitro by the experimental group is within the safe range of mammalian cells and has no obvious side effects, and GSK could prevent OVX-mediated bone mass loss by suppressing the formation of OCs via classical signaling pathways stimulated by RANKL, such as redox balance, NF-*κ*B, and MAPK, both in vivo and in vitro (Figure 8).

At the presence of RANKL stimulation, GSK may greatly reduce ROS levels and inhibit activities of NF-*κ*B and MAPK signaling pathways, thereby reducing the activity of NFATc1. Several factors affect intracellular redox states, including ROS levels and antioxidant enzyme levels [38]. ROS often accumulated during the procedure of OC formation induced by RANKL signals [39], and antioxidant enzymes are the important instruments of cells to scavenge excess ROS. For instance, HO-1 acted as a suppressive factor of OC differentiation via regulating the redox balance [40]. In addition, downregulation of GSR was found to activate the NF-*κ*B signaling pathway [41]. Furthermore, promoting the activity of GSR was reported to reduce the formation of OC mediated by estradiol [42]. Also CAT, a kind of antioxidant enzymes, led to the detoxification of hydrogen peroxide and block the ROS generation stimulated by RANKL [43]. Here, our data showed that GSK could attenuate the intracellular accumulation of ROS and increase the activity of antioxidant enzymes during the maturation of OCs. In summary, GSK suppressed ROS levels in OC differentiation via promoting intracellular capacity of antioxidant enzymes.

Accumulated evidence has shown that stimulation with RANKL could increase intracellular ROS levels that directly activate signal cascades of NF-*κ*B and MAPK signal transduction. NF-*κ*B signaling is crucial for the early differentiation of OC and sequentially activates downstream effector genes like c-Fos and NFATc1 [19, 39]. In transgenic mice, the loss of NF-*κ*B proteins directly impairs osteogenic effects and OC maturation [44, 45]. ROS could regulate the I*κ*B kinase degradation via oxidizing redox-dependent regulation of dynein light chain, followed by releasing of NF-*κ*B dimer and then allowing NFATc1 transfer into nucleus to initiate the differentiation of OCs [46]. In the present study, GSK could significantly prevent the degradation of I*κ*B-*α* induced by RNAKL and inhibit the phosphorylation of NF-*κ*B, suggesting a critical role in the suppression effect of GSK on the OC formation. MAPK signal proteins, such as ERK, JNK, and p38, have also been demonstrated a key role in osteoclastogenic function [47, 48]. Phosphorylation of ERK could promote the transcription of c-Fos, which leads to prolonging OC survival. Both stimulation of JNK and p38 also has been reported to induce the activation, differentiation, and fusion of OCs. Suppressing the activation of p-JNK and p-p38 could suspend OC formation and bone resorption induced by RANKL [49–51]. Moreover, high level of ROS, induced by RANKL, is reported to oxidize the MAPK signaling and lower the MAPK phosphatases [52]. Here, the present data demonstrated that GSK could suppress the phosphorylation of MAPK to prevent OCs formation. In addition, NFATc1 is the key signal transducer induced by RANKL and plays a critical role in OCs differentiation and proliferation [53, 54]. Furthermore, several multiple specific promoters are driven by NFATc1, such as cathepsin K, c-Fos, and DC-STAMP, which were essential for the OC maturation [55–57]. In the present study, our data demonstrated that NFATc1 activated by RANKL were significantly suppressed by GSK treatment. Taken together, GSK treatment could suppress the expression and activation of NFATc1 through NF-*κ*B and MAPK signaling pathways, thus downregulating specific promoter gene expression of TRAP, DC-STAMP, VATPs-D2, c-Fos, and CTSK *in vitro*.

Given that our *in vitro* data suggested the potential effects of GSK, we further studied the anti-OC effects of GSK on preventing bone loss and restoring the redox balance in OVX mice model. Ovariectomy female mouse model is widely used in studies focused on osteoporosis, because OVX could simulate the postmenopausal bone mass loss or osteoporosis. As expect, GSK attenuated OVX-mediated bone mass loss induced by low estradiol levels according to the results micro-CT and histological analysis. GSK treatment could suppress the number of TRAP-positive OCs in tibial bone which was consistent with in vitro results, while OVX-mediated oxidative stress, characterized by redox imbalance, could lead to osteopenia and further to osteoporosis [58]. Moreover, deficiency of estradiol could degrade the activities of antioxidant enzymes of tibial bone tissue in OVX [59]. The ratio of GSSG/GSH could suggest the status of redox balance, and normal ratio is essential for the survival of cells [60], as well as T-AOC, SOD, and CAT are the major effectors to act as free radical scavengers [41]. In the present study, our data showed that GSK could promote the activities of antioxidant enzymes in tibial bone and then attenuated the bone mass loss *in vivo*.

This study attempted to detect the precise inhibitory mechanisms by which GSK abrogated osteoclast-related osteoporosis. The present study revealed that GSK suppressed OCs formation by (1) downregulating ROS levels and (2) suppressing NFATc1, NF-*κ*B, and MAPK signaling pathways in vitro and inhibited OVX-induced bone loss in vivo. These findings suggested that GSK could be a potential therapeutic chemical molecule for treating the osteoclast-related osteoporosis especially postmenopausal osteoporosis. However, another goal for new osteoporosis drugs is to promote bone formation, and SGK1 was reported associated with osteoblastic formation according to previous studies [61, 62]. Thus, investigating the effects of GSK on the osteoblastic formation might be an interesting research area in the future. In addition, given that GSK is not the only selective inhibitor of SGK1, it is necessary to compare the effects of other kinds of SGK1 inhibitors on osteoclast differentiation in future studies.

In conclusion, the data of our study suggested that GSK inhibits osteoclastogenesis via suppressing multiple RANKL-induced signaling pathways at cellular level and improved the bone mass in an OVX animal model. These findings indicate that GSK might be a candidate therapeutic agent in treating bone loss-related diseases such as osteoporosis.

## Figures and Tables

**Table 1:** Primer sequences for real-time PCR.

Gene	Forward primer, 5′-3′	Reverse primer, 5′-3′
CTSK	CTTCCAATACGTGCAGCAGA	TCTTCAGGGCTTTCTCGTTC
c-Fos	CGGGTTTCAACGCCGACTA	TGGCACTAGAGACGGACAGAT
TRAP	CTGGAGTGCACGATGCCAGCGACA	TCCGTGCTCGGCGATGGACCAGA
VATPs-d2	AAGCCTTTGTTTGACGCTGT	TTCGATGCCTCTGTGAGATG
DC-STAMP	AAAACCCTTGGGCTGTTCTT	AATCATGGACGACTCCTTGG
NFATc1	CCGTTGCTTCCAGAAAATAACA	TGTGGGATGTGAACTCGGAA
Hmox1	AAGCCGAGAATGCTGAGTTCA	GCCGTGTAGATATGGTACAAGGA
Cat	AGCGACCAGATGAAGCAGTG	TCCGCTCTCTGTCAAAGTGTG
Gsr	GACACCTCTTCCTTCGACTACC	CCCAGCTTGTGACTCTCCAC
GAPDH	ACCCAGAAGACTGTGGATGG	CACATTGGGGGTAGGAACAC

## Data Availability

The data used to support the findings of this study are available from the corresponding author upon request.

## Abbreviations

OP: Osteoporosis
OC: Osteoclast
ROS: Reactive oxygen species
GSK: GSK
RANKL: Receptor activator of nuclear-*κ*B ligand
NFATc1: Nuclear factor of activated T cells c1
TRAF6: TNF receptor-associated factor 6
HO-1: Heme-oxygenase-1
SOD: Superoxide dismutase
SGK1: Serum- and glucocorticoid-inducible kinase 1
BMMs: Bone marrow macrophages
GSSG: Oxidized glutathione
GSH: Glutathione
CAT: Catalase
T-AOC: Total antioxidants.

## Supplementary Materials

Supplementary Materials Figure S1. Hematoxylin-eosin (H&E) staining of the major organs derived from mice at the end of treatment. The heart, liver, spleen, lung, and kidney tissue slices, scale bar: 200 *μ*m.
