# Supplementary figures and images for: GSK 650394 Inhibits Osteoclasts Differentiation and Prevents Bone Loss via Promoting the Activities of Antioxidant Enzymes In Vitro and In Vivo
Source: Oxid Med Cell Longev. 2022 Sep 17;2022:3458560. doi: 10.1155/2022/3458560 (PMC9509242; doi:10.1155/2022/3458560)

Figure S1


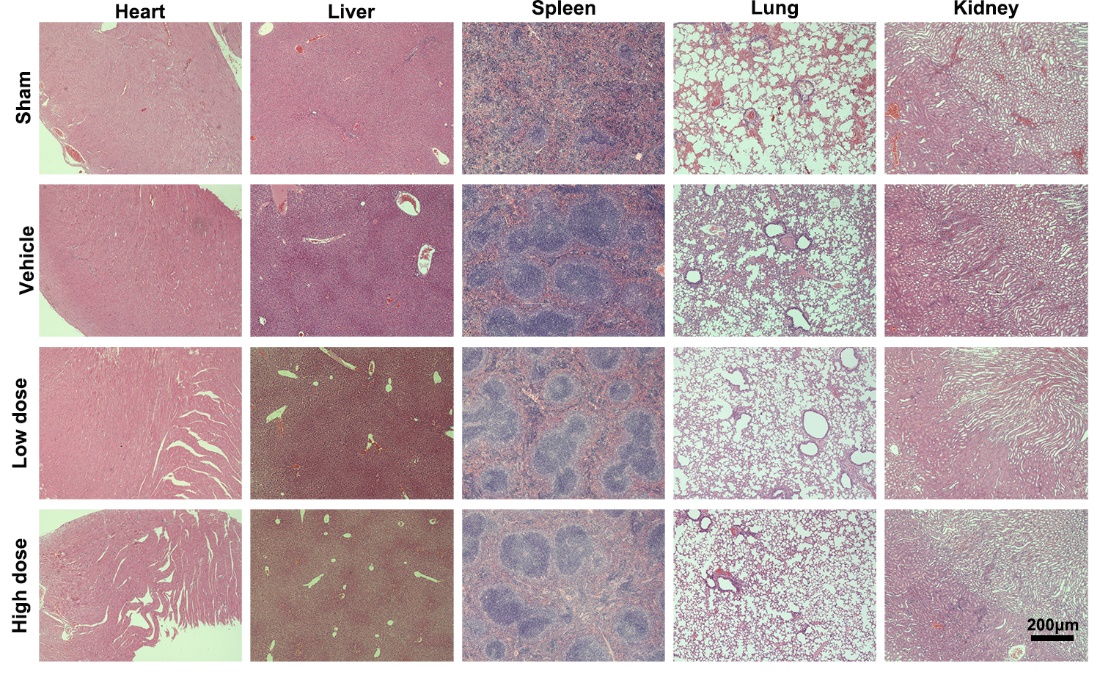

Supplement: Supplementary Materials — Figure S1. Hematoxylin-eosin (H&E) staining of the major organs derived from mice at the end of treatment. The heart, liver, spleen, lung, and kidney tissue slices, scale bar: 200 μm. [file 3458560.f1.docx]
